# The role of neuronal plasticity in cervical spondylotic myelopathy surgery: functional assessment and prognostic implication

**DOI:** 10.1007/s10143-023-02062-9

**Published:** 2023-06-26

**Authors:** Lapo Bonosi, Sofia Musso, Luigi Maria Cusimano, Massimiliano Porzio, Evier Andrea Giovannini, Umberto Emanuele Benigno, Giuseppe Roberto Giammalva, Rosa Maria Gerardi, Lara Brunasso, Roberta Costanzo, Federica Paolini, Andrea Sciortino, Benedetta Maria Campisi, Kevin Giardina, Gianluca Scalia, Domenico Gerardo Iacopino, Rosario Maugeri

**Affiliations:** 1 Neurosurgical Clinic, AOUP “Paolo Giaccone”, Post Graduate Residency Program in NeurologiSurgery, Department of Biomedicine Neurosciences and Advanced Diagnostics, School of Medicine, University of Palermo, 90127 Palermo, Italy; 2 Department of Neurosurgery, ARNAS Garibaldi, P.O. Garibaldi Nesima, 95122 Catania, Italy

**Keywords:** Cervical myelopathy, Functional reorganization, White matter tracts, fMRI, DTI, Surgery

## Abstract

Cervical spondylotic myelopathy (CSM) is a degenerative disease representing the most common spinal cord disorder in the adult population. It is characterized by chronic compression leading to neurological dysfunction due to static and dynamic injury of the spinal cord in cervical spine. These insidious damage mechanisms can result in the reorganization of cortical and subcortical areas. The cerebral cortex can reorganize due to spinal cord injury and may play a role in preserving neurological function. To date, the gold standard treatment of cervical myelopathy is surgery, comprising anterior, posterior, and combined approaches. However, the complex physiologic recovery processes involving cortical and subcortical neural reorganization following surgery are still inadequately understood. It has been demonstrated that diffusion MRI and functional imaging and techniques, such as transcranial magnetic stimulation (TMS) or functional magnetic resonance imaging (fMRI), can provide new insights into the diagnosis and prognosis of CSM. This review aims to shed light on the state-of-the-art regarding the pattern of cortical and subcortical areas reorganization and recovery before and after surgery in CSM patients, underlighting the critical role of neuroplasticity.

## Introduction

Cervical spondylotic myelopathy (CSM) is a comprehensive definition used to describe a complex of age-related and progressive conditions affecting the cervical spine structures, ultimately leading to chronic compression of the cervical spinal cord [1]. It is adults’ leading cause of spinal cord injury [2]. Based on data for the general population, CSM affects approximately 10% of patients over 50 years old. Notably, even though more than 50% of individuals show radiologic evidence of spondylosis of the cervical spine tract, only a fraction of the patients tends to develop clinical signs [3]. Degeneration involves the intervertebral disc and the capsule-ligamentous and bony compartments of the cervical spine. These pathological modifications lead to the spinal canal and foramen’s narrowing, eventually resulting in neural structure compression [2, 4, 5]. Long-term compression of the cervical spinal cord can cause the degeneration of the anterior horn and motor neurons, even the lateral and posterior funiculus axons demyelination.

Typical findings include limb numbness and bilateral fine motor deficits with loss of dexterity, hemi- or quadriparesis, ataxic gait, sphincter disturbances, hyperreflexia, and clonus [6]. Treatments include conservative management, such as neck immobilization, pharmacologic therapies, lifestyle modifications, physical modalities, and surgical options [7].

The goal of surgery is the expansion of the spinal canal to provide a decompression, improve spinal cord morphology, and achieve a successful fusion, thus preventing the development of late deformity.

**Fig. 1:**
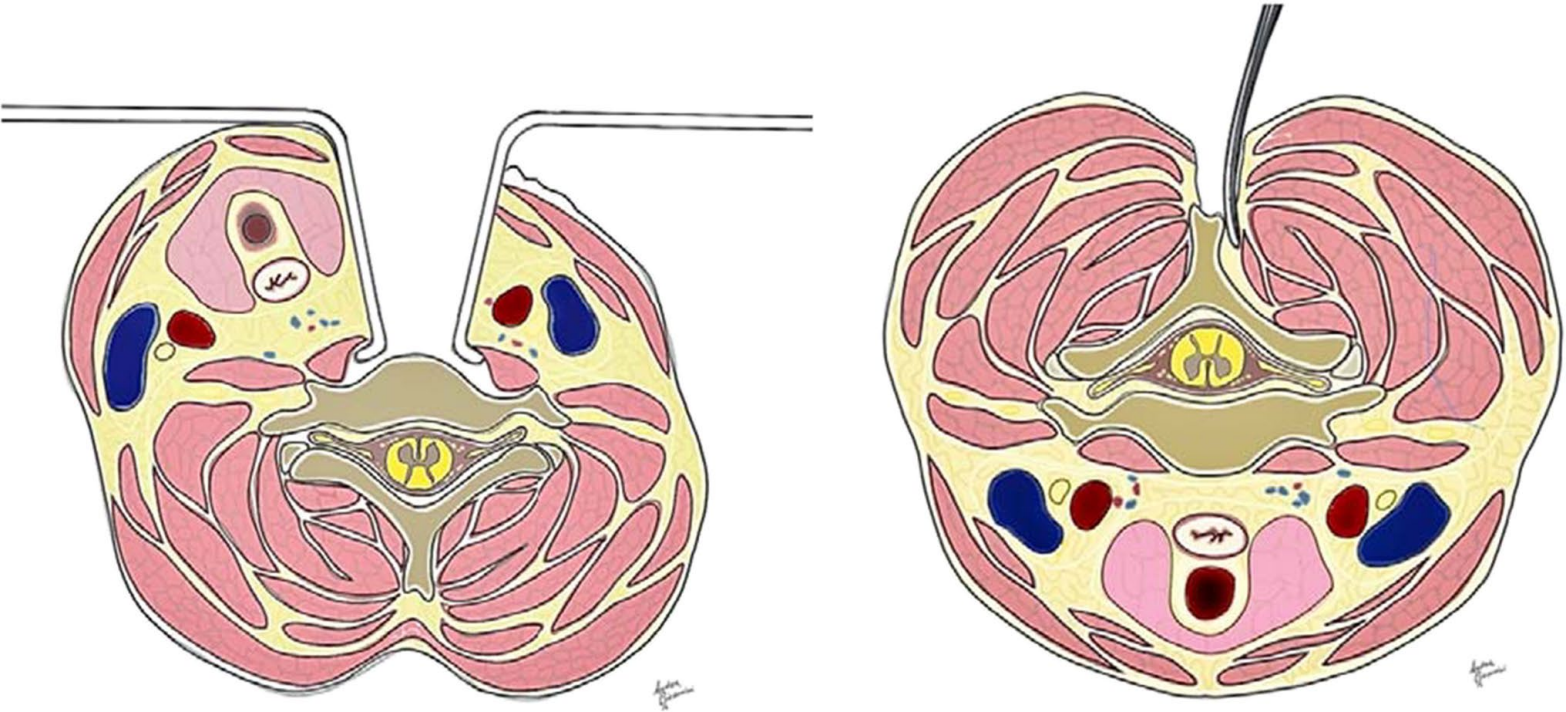
**A–B** Illustrations of anterior and posterior surgical corridors for cervical myelopathy

There are many options in the operating room, including anterior cervical discectomy and fusion (ACDF), which represents one of the most commonly performed surgical procedure in CSM, firstly described by Robinson and Smith in 1955 and by Cloward in 1958 [8, 9] (Fig. 1A); corpectomy [10, 11]; laminoplasty that allows for avoiding the implant of vertebral screws [12]; and laminectomy (with or without fusion), the procedure of choice in multiple-level compression in patients with preserved cervical lordosis. The latter especially indicated in elderly patients, where comorbidities increase the operative risk [13–15] (Fig. 1B).

Finally, it is possible to perform combined approach, particularly indicated for patients with severe kyphotic angulation necessitating decompression of 3 or more levels [16].

To date, decompressive surgery remains the most effective long-term treatment for this pathology, although the decision of which type of approach use and when to perform such a procedure remains challenging [17].

Several prognostic factors could affect the outcome after surgical management [18]. The most important prognostic factors are duration of symptoms, preoperative neurological status, the effective diameter of the canal, number of compression levels, intrinsic cord alterations (assessed from pre-operative MRI study), and other clinic-radiological features [3, 19–22]. In recent years, novel techniques have been applied to investigate central nervous system (CNS) pathology [23, 24]. 

For instance, it has been shown that diffusion weighted imaging (DWI), tractography, fMRI, and other functional techniques are more valuable than routine MRI scans for diagnosis and predicting outcomes in CSM patients [25, 26]. DTI-tractography and diffusion MRI (dMRI) techniques in general allow the mapping of neural connections by tracking water movement across axons while simultaneously measuring important metrics like fractional anisotropy (FA), mean diffusivity (MD), axial diffusivity (AD), and radial diffusion (RD) [27, 28]. FA quantifies how much directional coherence exists between tissues — giving an insight into nerve tract connectivity quality and correlating with tissue integrity [29]. Instead, MD values in brain regions indicate the water diffusion degree, and higher values are often associated to brain disease. Conversely, AD and RD reflects other features related to axonal structure and integrity [30, 31]. On the other hand, functional MRI-based imaging technique is based on the analysis of the BOLD (blood oxygen level-dependent) signal, which in turn estimates changes in blood oxygenation in response to neural activity or at rest (the so-called resting state fMRI or rsfMRI) [32]. Particularly important are the spatial and temporal resolution of the signal and the VOA, which need to be carefully known and interpreted [33–35]. Finally, TMS represents a noninvasive brain stimulation technique used to induce excitability/inhibition changes in the cerebral cortex through a coil that generates a magnetic field. It has become a fundamental tool for diagnostic purposes to mapping brain functioning in humans (the so called navigated TMS, or nTMS) and for therapeutic ones to induce neuroplasticity phenomena (repetitive TMS, or rTMS) [36]. Several TMS stimulation paradigms exist, such as short interval cortical inhibition (SICI), intracortical facilitation (ICF), long interval cortical inhibition (LICI), and paired associative stimulation (PAS), each with its own peculiarities and indications [37–39].

Despite these advanced tools, only a few determinants of functional recovery have been defined [40, 41]. The complex physiologic recovery processes involving cortical and subcortical neural reorganization following surgery are still inadequately understood. To assess the functional recovery of the central nervous system (CNS) following CSM surgery, many diagnostic imaging techniques, including fMRI, PET, TMS, diffusion tensor imaging tractography, and other functional studies, have been proposed [40–42]. Neuronal plasticity affects structural and functional reorganization of either brain or spinal cord fiber tracts, allowing, over time, for compensation of the previously established neurological deficits [43, 44]. To date, there are still no validated scores analyzing pre- and postoperative supraspinal functional reorganization, with the goal of stratifying patients prognostically, trying to understand which variables and factors are involved in neuroplasticity and how they influence the recovery process.

This review aims to evaluate the state-of-the-art about pattern of cortical reorganization and recovery of the CNS after surgery in CSM patients and to find possible prognostic factors associated with the types of cortical reorganization.

## Materials and methods

### Search of the literature

**Fig. 2:**
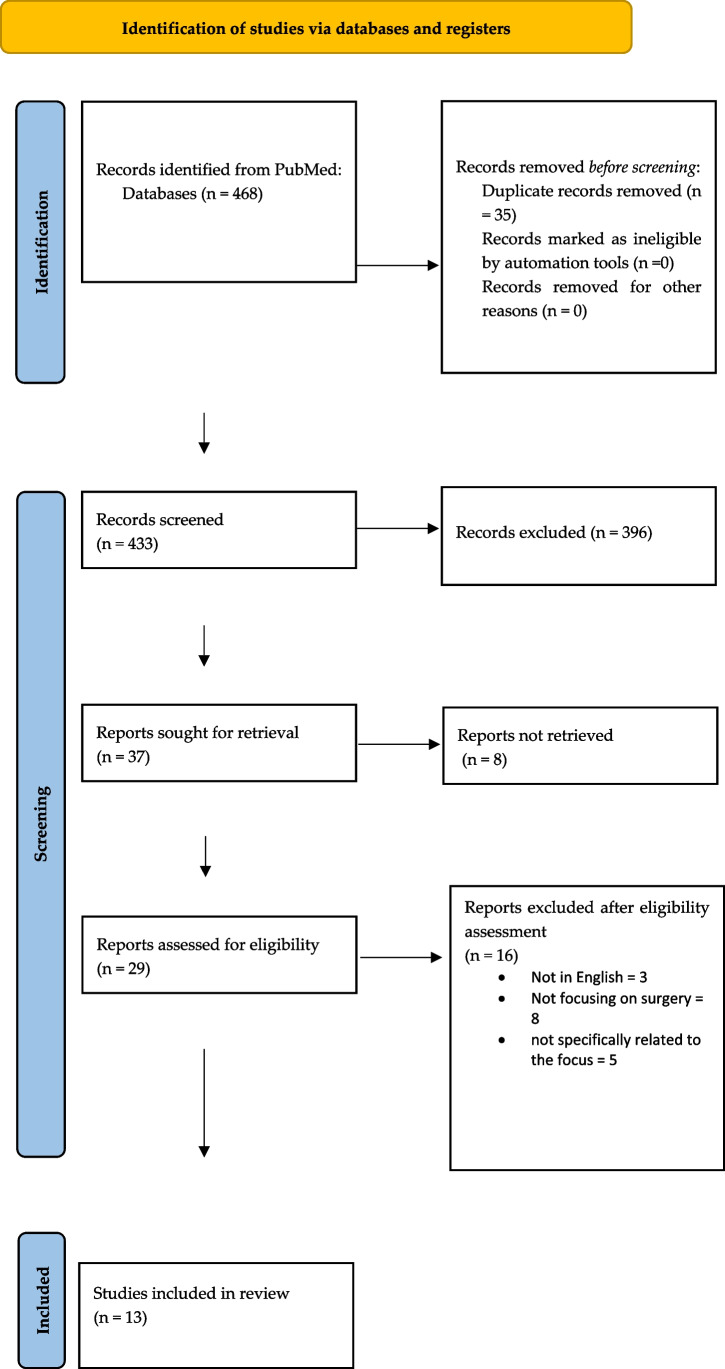
PRISMA flow chart of selection process

Preferred reporting items for systematic reviews and meta-analyses guidelines (PRISMA) were followed to conduct and report this systematic literature review (Fig. 2) [45]. We performed a broad systematic literature search in Pubmed for all studies investigating neuroplasticity associated to cortical reorganization in patients with cervical myelopathy undergoing surgery. We searched for studies published up to the 17th of May 2022 without backward limits, using the following MeSH and free text terms “cervical myelopathy”, “functional reorganization”, “white matter tracts”, “FMRI”, “tractography”, “cortical reorganization”, and “surgery”, combined using Boolean operator “AND”. To avoid the potential omission of relevant studies, we also manually screened reference lists of articles included and previous systematic reviews and meta-analyses regarding cervical myelopathy, surgery, and cortical functional reorganization and neuroplasticity. Duplicate articles were eliminated using Microsoft Excel 16.37.

### Study selection

The research strategy initially relied on title and abstract analysis. The article’s full text was retrieved for further investigation if the title and abstract met the inclusion criteria. The data collection process was conducted without using any automated tools. No ethical approval was required for this study.

### Eligibility criteria

The articles were selected according to the following inclusion criteria:

- Full article in English
- Case report, case series, retrospective study, and prospective study
- Patients age ≥ 18
- Patients affected by cervical myelopathy (defined clinically by standardized scales such as the mJOA or radiologically, as an area of hyperintensity at the level of the medullary cord in T2-weighted MRI sequences).
- Patients treated by surgery with subsequent investigation of cortical functional reorganization and neuronal plasticity.

Exclusion criteria:

- Articles not in English
- Editorials, books, systematic reviews, and meta-analysis
- Patients age < 18
- Patients treated without surgery.
- Studies not evaluating cortical neuronal plasticity in the pre- and post-operative period.
- Studies in a preclinical phase (animal study or in vitro study)

### Data extraction

According to the criteria above, all articles were identified by two reviewers (L.C and M.P). In case of a discrepancy, a third author (L.B) arbitrated until a consensus among the authors was reached.

The extracted data included the following: publication’s year, author, patients’ number, country, study design, postoperative clinical improvement (assessing through post-op mJOA score), methodic, area of interest, postoperative vs. preoperative comparison, post-operative vs. control comparison, others, and analyzed value.

### Statistical analysis

Microsoft Excel – 2022 and Prism – GraphPad 2022 have been used for statistical analyses. Means and SDs or medians and ranges were calculated for continuous variables depending on the distribution of the variable. Frequencies and percentages were calculated for nominal variables. A 2-sided *χ*^2^ test or an independent 2-tailed and unpaired *t* test was performed to test differences between patients and control group or preop mJOA and postop mJOA. Only results with a *p* value < 0.05 were considered statistically significant.

## Results

### Data selection

Our initial research through Pubmed identified a total of 468 articles. We excluded 35 duplicated articles, then performed further screening based on title and abstract reading, eliminating 396 articles.

Finally, after a full-text reading and a detailed examination, we included 13 studies in our systematic review, according to PRISMA flow diagram inclusion criteria.

The characteristics of included articles are shown in Table 1. Data extracted were the number of patients, age, gender, the control group characteristics, pre-, and postoperative mJOA scores, methods used, investigated area, differences between control group, pre-, and postoperative patients.

**Table 1:** Reported data from the reviewed studies and results

*N*	Year	First author	Country	Study design	Pt	N° C	Mean age	Male pt	mJOA preop	mJOA postop	mJOA postop vs. preop	Methodic	Value	Regions of interest (ROIs)	Postop vs. preop comparison	Preop vs. C comparison	Postop vs. c comparison	Others
1	2019	Peng et al. [46],	China	Retrospective	43	43	49.07 ± 6.73	27	11.16 ± 2.21	Increased	Improved	fMRI	FC	Visual cortex		Increased		/
													FC	Motor area	Decreased	Decreased	Decreased	/
													FC	Sensory pathway			Increased	/
2	2010	Duggal et al. [47],	Canada	prospective	12	10	49.6 ± 12.8	8	11.8 + 2.6	14.3 ± 2.5	Improved	fMRI	VOA	Sensory area	Increased	Lower		/
													VOA	Motor area	Increased	Increased	Increased	/
3	2010	Tam et al. [41],	Canada	Case report	1	/	37	1	N/A*	Improved by 33%	Improved	fMRI	VOA	Contralateral motor area, cerebellum	Increased	/	/	/
4	2018	Sawada et al. [48],	Japan	Cross-sectional	6	5	48,16	3	12.50 ± 2.47	14.75 ± 2.04	Improved	fMRI	VOA	Motor area, acc	Decreased	Increased		/
5	2007	Holly et al. [40],	California USA	Prospective	4	5	58,25	1	13.5	16.5	Improved	fMRI	VOA	Contralateral motor area, dorsal premotor	Decreased	Increased	Similar	/
6	2016	Bhagavatula et al. [43],	India	Prospective	17	12	51.76 ± 10.67	16	10.71 ± 2.64	14.88 ± 2.49	Improved	fMRI	VOA	Contralateral sensory and motor area	Decreased	Increased	Increased	/
													VOA	Cerebellum	Lower	Increased	Increased	/
7	2016	Aleksanderek et al. [49],	Canada	Prospective	28	10	51.55 ± 10,5	21	12.85 ± 1.35	15.185 ± 1.55	Improved	fMRI	VOA	Sensory area	increased	Increased		/
													VOA	Motor area	Decreased	Increased		Increased in mild vs. moderate CSM
8	2018	Ryan et al. [50],	Canada	Prospective	22	10	50 ± 10.9	19	13 ± 3	16 ± 2	Improved	fMRI	VOA	Contralateral sensory area	Decreased	Decreased	Decreased	/
													VOA	Contralateral motor area	Decreased	Increased	Increased	Contralateral M1 VOA at baseline was significantly correlated with mJOA scores at baseline and 6 months after surgery
9	2015	Green et al. [51],	Singapore	Prospective	24	15	58.2 ± 11.5	16	12.7 ± 2.81	13.81 ± 3.1	Improved	TMS	MEP	Motor area	Decreased	Increased	Similar	/
10	2020	Paliwal et al. [52],	Okhlahoma USA	Prospective	13	9	55.9 ± 14.0	9	14.5 ± 2.0	16.10 ± 1.89	Improved	MRI	MTR	Rubrospinal and spinocerebellar tract				Improvement of postop mJOA was associated with pre-op mean MTR across all levels and at the region of compression
11	2012	Nakamura et al. [42],	Japan	Prospective	16	4	64.6	15	N/A*	N/A	N/A	DTI	FT ratio	Number of fibers at the compressed level/number of fibers at the C-2 level	Increased	Similar	/	Positive correlation between preop FT ratio and recovery rate
12	2011	Woo Lee et al. [53],	Korea	Prospective	20	20	49.6	13	10.7	5 pt with intact FT: 80% improvement after surgery. 5 pt with interrupted FT:20% improvement after surgery	Improved	DTI	FT ratio	FT passing through ROI at both C1 and C7 level				Postop neurologic improvement was connected to patients with intact fiber tractography and not to those with interrupted fiber tractography
													FA			Decreased		/
													ADC			Increased		/
13	2019	Bhosale et al. [54],	India	Prospective	30	/	N/A	29	N/A*	Improved	Improved	DTI	FA		Increased	Decreased		FA and ACD correlated to postop neurological status

Abbreviations: *mJOA* Modified Japanese Orthopedic Association score; *C* control; *FC* functional connectivity; *VOA* volume of activation; *Pt* patient; *CSM* cervical spondylotic myelopathy; *FA* fractional anisotropy; *FT* fiber tract; *ADC* apparent diffusion coefficient; *MEP* motor evoked potentials; *MTR* magnetization transfer ratio; *TMS* transcranial magnetic stimulation

*The diagnosis of cervical myelopathy was made based on the patient’s symptoms (without reporting the preoperative mJOA score) and/or the radiological finding of intensity signal changes on MRI at the level of the compressed spinal cord

### Patient’s demographic data and study characteristics

We have analyzed a total number of 240 patients, which mean age was 51.97 ± 6.84 years. This cohort has a male prevalence compared to female (74.16% vs. 25.83%).

As shown in Table 1, among our selected studies, three papers analyzed the role of DTI in cervical myelopathy, eight papers fMRI, one article on other tools associated with MRI, and one paper on transcranial magnetic stimulation to evaluate MEP. Eleven of all the works included in our review had a study design including a control group cohort.

Clinical and demographic data of our selected cohort are also reported in Table 1.

Data from patients who undergone to fMRI had shown that, when motor area was chosen as ROI, there was a reduction of postoperative FC of approximately the 87.5% compared to preoperative; in all cases (100%), it has been shown a higher value for preoperative FC in comparison to control group. When ROI was put on the sensory area, it was enlightened that postoperative VOA had a lower value than preoperative VOA in the 50% of cases and preoperative VOA had a lower value than controls in the 50% cases.

In patients who have undergone DTI-tractography, it has been shown in one study that postoperative neurological improvement was more common in patients with intact fibers, in another study that postoperative mJOA tends to a positive correlation to postoperative FT and in the third study that preoperative FA and ADC values have a statistically significant correlation to postoperative mJOA.

In all studies evaluating the neurological status by mJOA score, a statistically significant difference has been shown between preoperative mJOA and post-operative mJOA, underling a beneficial effect of decompressive surgery (*p* < 0.001).

## Discussion

### The concept of neuroplasticity

CSM is a debilitating condition characterized by neurological impairment due to static and dynamic injury of the spinal cord in the cervical spine [55]. Chronic spinal cord compression produces necrosis and demyelination of gray and white matter, resulting in progressive neurological impairment associated with motor, sensory, and autonomic disability [1].

**Fig. 3:**
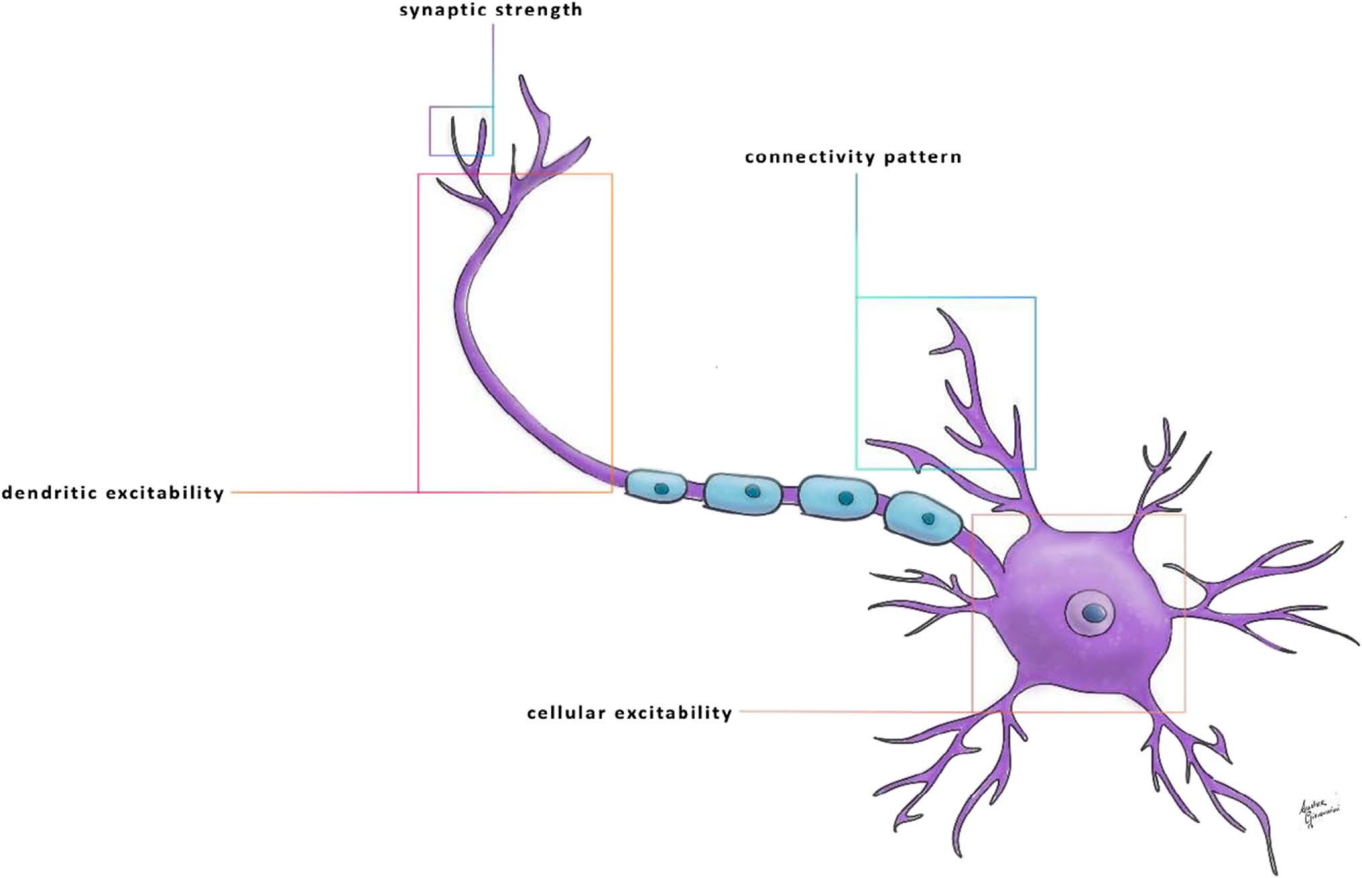
Schematic representation of the neuronal plasticity and cellular processes involved. Schematic representation of the neuronal plasticity mechanisms: enhanced synaptic strength; increased dendritic excitability facilitates synaptic integration and neuronal firing; enhanced cellular excitability results in a lower threshold for potential action generation; altered connectivity pattern results in neuronal network modulation and increased neuronal activity

In this context, it has been demonstrated that neural plasticity of the cortical network allows for the minimization of functional impairment. It can occur via synaptic modification of preexisting connections or by developing new circuitry [56–58]. Neuronal plasticity allows neurons in the brain and spinal cord to compensate for injury by adjusting their activities or structures in response to new situations [59]. The cellular correlates underlying neuronal plasticity involve morphological and functional adaptations in synapses, dendrites, and axons. These mechanisms result in a change in the input–output function of the neural network and, therefore, in a modification of information processing [60, 61] (Fig. 3).

Contrary to what is postulated by the theory of localizationism, whereby each part of the brain is dedicated to a specific function and operates independently of the other portions, the concept of neuronal plasticity is based on the idea that the brain and spinal cord, when faced with events disrupting their normal physiology, can build up or adapt themselves to variable or persistent demands to limit the damage and safeguard the injured function [62, 63]. Cornerstones of neuroplasticity are modularity, redundancy, and distributed processing [64]. Modularity is the organization of a system into modules. Each module performs a discrete function, and if one module is eliminated selectively, the remaining modules can sustain the global role of the system. Otherwise, redundancy ensures the persistence of a function even after the brain’s partial destruction, thus indicating that distinctly different elements may sustain the same function [65, 66]. From an anatomophysiological point of view, processes involving neural plasticity include neurogenesis, cell migration, changes in neuronal excitability and neurotransmission, the generation of new connections, and modification of existing ones [67, 68].

### Tools for evaluating cortical functional reorganization

The dorsal column of the spinal cord is composed of sensory afferent fibers. Due to an injury, the section of this pathway in the cervical spinal cord can deactivate much of the primary somatosensory cortex and other areas. Following this injury, these affected areas can go through a reorganization to ensure a functional recovery by creating new neuronal circuitry [69]. Over the years, several studies focused on the concept of neural plasticity following cervical injury, the imaging techniques to evaluate it, and the relation between preoperative and postoperative imaging findings and the functional recovery of patients after surgery.

For instance, it is possible to employ functional MRI (fMRI) to evaluate neural plasticity. In particular, the differentiation due to acute spinal cord injury causes an immediate change of wide cortical networks that can be noticed on fMRI, which shows the expansion of M1 representation and the increased activation of supplementary motor areas as the result of the cerebral reorganization [40, 70].

Duggal et al. [47] compared preoperative and postoperative brain fMRI to characterize the changes occurring in cortical activation after surgical decompression: in preoperative patients, the volume of activation (VOA) was greatest compared to controls within the precentral gyrus, while VOA was greater after surgical decompression, a characteristic which may represent increased recruitment within the primary motor cortex in patients with cervical myelopathy; instead, within the postcentral gyrus, VOA was greater in controls, which may reflect a degree of local cortical atrophy. The degree of VOA at fMRI is still a matter of debate, since it plays a divisive role, marking a difference between various included studies’ results; it seems clear that there are studies in which VOA is increased, between preoperative and postoperative evaluation, and others where is decrease. Sawada et al. [48] have tried to explain this contradiction referring to the absence of an in-depth study of the relationship between task difficulty and recruited brain region, used in the assessment of VOA. Bhagavatula et al. [43] switch the focus on the heterogeneity of patients with CSM which present with different degrees of spinal cord impairment and they reflected on how the extent of synaptic transmission and reorganization depend on the time elapsed since the initial insult and the heterogeneity of residual spinal cord atrophy. All these topics suggest that there is still no simple and unambiguous way to interpret these data and further studies on this topic are needed.

From a structural connectivity point of view, DTI-tractography is an MRI technique that allows the noninvasive measurement of the translational motion of water, providing information such as the FA in different tissues and measuring their integrity. Several studies reported changes in parameters in patients affected by cervical myelopathy compared to healthy controls, especially in the FA values, which seem to be strictly related to recovery rates in patients affected by cervical myelopathy, while the high signal intensity of the spinal cord on T2WI does not. Thus, FA, and DTI-tractography, can be used as objective prognostic factors to predict the patient clinical outcome and the rate of functional recovery after decompression surgery [42, 54, 71]. However, DTI-tractography has some intrinsic limitations. First, it is not sensitive to the complex architecture of cortical white matter, particularly it cannot directly image multiple fiber orientations within a single voxel; the reason for this is that the tensor model approaches fiber orientation with an ellipsoid shape. In a region where fibers cross, the orientation estimation of the tensor model will approach a sphere and thus cannot capture the orientation of two separate fibers [72]. To overcome this drawback various algorithms have been proposed, including constrained spherical deconvolution (CSD) [73], spherical-deconvolution informed filtering of tractograms (SIFT) [74], or multi-shell multi-tissue CSD (MSMT) [75]

Another possible technique is magnetic resonance spectroscopy (MRS), which can detect metabolites and study tissue biochemistry in vivo. In CSM patients, MRS can identify a decreasing inN-acetylaspartate (NAA) to creatine (Cr) ratio in the primary motor cortex after injury. These alterations suggest neuronal impairment or altered energy metabolism and have proven to be strictly related to changes in mJOA scores after surgery [76].

DTI and MRS can be used in combination to provide a stronger correlation between the patients’ symptoms, assessed through the mJOA score, and the degree of neurological impairment. Thanks to the evaluation of DTI parameters (e.g., FA) and MRS biomarkers (e.g., Cho/NAA ratio), it is possible to assess the microstructural and metabolic patterns following the cervical injury and better define the post-surgical functional status [77].

Recently, navigated transcranial magnetic stimulation (nTMS) has proven to be a valuable tool in characterizing functional impairment in patients affected by cervical myelopathy. Indeed, some neurophysiological measurements, such as the corticospinal excitability, rate of inhibition, and motor area behavior, are related to the symptomatology and the clinical course. The detection of the degree of compensatory reorganization given by these parameters can be therefore used to stratify patients in terms of risk for further neurological deterioration [70]. nTMS can also be used after decompression to demonstrate the compensatory expansion of motor cortex, to identify patient potentially recovery and the correct rehabilitative program after surgery [51].

The articles included in our review point out the remarkable usefulness of such techniques for diagnostic and, mostly, prognostic purposes due the opportunity to obtain information about the functional aspect of the injured cervical spinal cord, and the cortico-subcortical reorganization of particular brain areas. However, even though they are currently employed in the clinical practice, they do not belong to standardized diagnostic algorithms and are a prerogative of few specialized units.

### The effect of surgery in cortical areas reorganization in cervical myelopathy

It has been observed that neck disability and symptom severity in cervical myelopathy can be related to brain alterations in both cortical microstructure and functional connectivity [78], such as the decrease in cortical thickness and the increase in functional connectivity between primary sensorimotor and supplemental motor or sensory regions. Consistent with these observations, Wang et al. [57] observed significant alterations in white matter tracts connecting primary motor and sensory cortices using diffusion spectrum imaging (DSI). This method, unlike classical DTI-tractography, is able, through special acquisition protocols and post-processing software, to detect the orientation of multiple fibers within a voxel, allowing the delineation of fibers that cross and touch in the brain. This technique is based on the study of the probability density function (PDF) that for each voxel specifies the 3D distribution of microscopic spin displacements visible in MR that it contains [79]. Given the ability to accurately display the structural changes of fiber tracts, DTI based on DSI has already been used in some neurodegenerative and mental disorders, with promising results [80, 81].

Aleksanderek and colleagues [49] compared the functional reorganization in the primary motor cortex in patients affected by mild and moderate CSM by using the MR spectroscopy. They found that, before surgery, the NAA/Cr ratio was lower in patients with mild CSM, compared to healthy control patients and subjects with moderate CSM. Following surgery, both the groups demonstrated a functional improvement, and six months after surgery, the NAA/Cr ratio decreased significantly in patients affected by moderate CSM. The neurological recovery observed can thus be explained by the mitochondrial and synaptic dysfunction, evidenced by low levels of NAA/Cr, which represent the primary trig for cortical reorganization.

Cortical changes occurring after surgery were also evaluated by Bhagavatula et al. [43] by using the blood oxygen level-dependent (BOLD) functional MRI (fMRI): after CSM, to compensate for motor weakness and loss of dexterity, there is over-recruitment of sensorimotor cortices (left precentral gyrus) documented by an VOA, compared to the control group. Postoperatively, in response to surgery, the cortical reorganization is demonstrated by activating and recruiting other areas, such as the premotor and supplementary motor areas. This recruitment can be already observed six months after surgery and can be relevant for the maintenance of function after injury and in the recovery process itself [50].

Functional reorganization is typical in CSM patients localized to sensorimotor, regulatory, and visual processing regions [82]. Indeed, after CSM the sensory components transmitted to the thalamus can decrease, thus leading to alterations in thalamus-cortex circuits. Through fMRI, it is possible to study the alterations in functional connectivity values between the thalamus and the cortex. The abnormal values observed after the cervical injury (increased compared to healthy controls) demonstrate that spinal damage has an impact on brain activity and can be used as biomarkers to assess neuronal damage and predict patient outcomes; moreover, the altered functional connectivity observed in post-surgery patients (which is decreased compared to healthy controls) is suggestive for the adaptive changes occurring after decompression [46]. Also, the brainstem and the cerebellum lobes, evaluated through fMRI, showed to play a relevant role in the pathway of functional brain reorganization in patients affected by cervical injury [83].

### Prognostic factors related to neural plasticity in CSM

Strategies to predict neurological recovery and clinical outcomes following decompression surgery in patients affected by CSM are needed. The current prediction is based on multiple factors: age, duration of symptoms, preoperative neurological status, and radiological findings, such as signs of instability or T2 high-intensity signals on MRI [84, 85]; on the contrary, the type of approach chosen by the surgeon, both ventral or dorsal, does not seem to affect the prognosis [86]. However, a prediction based on these factors remains subjective and not quantitative [87].

In patients affected by CSM, functional alterations can be observed in the visual cortices through fMRI and these alterations are strictly related to visual acuity. Zhou et al. [88] observed that patients show higher bidirectional effective connectivity between the secondary visual cortex and the cerebellum in CSM patients, and this increase seems to be related to the prognosis. Consequently, structural alterations and adaptive changes in patients suffering from myelopathy play a crucial role in neuropathology.

To define the relation between functional reorganization and clinical outcome, thus identifying prognostic markers, the functional connectivity (FC), a technique used to identify spatial patterns of coherent BOLD activity, can be used. According to literature, an increased FC between visual associated brain regions and the cerebellum is related to altered visual function and impaired motor function, therefore negatively related to JOA scores in patients affected by CSM [89]; accordingly, FC may act as a potential biomarker for postoperative gain and potential recovery [90]. The association between FC and prognosis could also be explored via support vector regression (SVR), using preoperative FC as features and JOA recovery rate as labels. Predicting CSM-related outcomes through machine learning techniques is not yet robust enough to be used in clinical practice, and so further studies are needed [91].

Another fMRI technique used to predict the prognosis of patients is the multivariate pattern analysis, which evaluates the static amplitude of low-frequency fluctuation (sALFF) and the dynamic amplitude of low-frequency fluctuation (dALEF). Brain regions that successfully predicted the clinical outcome were mainly located at the frontal cortices for sALFF and frontal cortices, left insular, and posterior lobe of the cerebellum for dALFF. Therefore, the functional alterations documented by the static and dynamic ALFF are related to patient’s clinical outcome and can be employed to determine prognostic biomarkers for cervical myelopathy [92].

Moreover, CSM patients exhibit a higher ALFF within left motor cortex and bilateral superior frontal gyrus and lower zALFF within right precuneus and calcarine; the functional status of M1 contributes to the severity of CSM, and thus the M1 zALFF can be considered a valuable predictor of the prognosis in CSM patients after decompression surgery, given that patients with more severe symptoms show this pattern on fMRI [93]. Moreover, preoperatively increased ALFF decreases after surgery in the primary sensorimotor cortex and visual cortex [87].

Craciunas et al. [94] found that the postoperative clinical status is strictly associated with preoperative levels of specific metabolites across M1 and the cerebellum. Specifically, preoperative levels of myo-inositol and glutamate–glutamine in the cerebellum were associated with the extent of lower extremity disability. In contrast, higher levels of the upper-extremity sensorimotor function were related to higher levels of NAA and glutamate–glutamine in the left M1. These findings suggest that patients with less neuroinflammation and neuronal metabolic depression have a higher potential for functional reorganization.

## Limitations

Given the studies heterogeneity and the small number of samples examined, the picture rendered to the reader appears to be a general overview of the functional and structural connectivity imaging and stimulation techniques that are increasingly being employed in this setting and their potential. In this context, our study presents some limitations. First, the paucity of studies in this regard and the small sample size examined do not allow generalization of the results, further leading to the difficulty in translating the results obtained into daily clinical practice. Secondly, the studies included in this review consider different methods in assessing the functional reorganization of cortical networks, making interpreting results and their applicability for prognostic purposes difficult. Third, another limitation is the nonunique definition of cervical myelopathy. In fact, the diagnosis of CSM is made using clinical criteria, radiological criteria, or both. To overcome this obstacle, the use of standardized clinico-radiological scores would certainly help to quantitatively implement the inherently qualitative definition of cervical myelopathy. Finally, further longitudinal studies appear to be necessary to evaluate the effect of decompression surgery on alterations of dynamic connectomics of brain networks. Moreover, the lack of standardized acquisition protocols results in a potential risk of bias in the analysis of results obtained even with identical tools. This last point can only be overcome by introducing these techniques into clinical practice and routine evaluation of the patient with cervical myelopathy, adopting standardized algorithms to improve the collection and analysis of the data obtained also from different centers.

## Conclusions

CSM is a subtle and debilitating condition resulting in various neurological deficits. Neural plasticity of the cortical network allows functional impairment to be minimized. Several imaging modalities exist to assess the functional status and postoperative reorganization of the neural network in patients affected by CSM. These techniques could be helpful in more accurately predicting the outcome of patients undergoing cervical surgery. Our review represents a first effort to unify the results obtained so far in this new and still under-explored field. Much ground remains to be covered in understanding the precise role of each of these tools, the data they provide, and their usefulness in clinical practice.

Nonetheless, surveying the relationship between CSM and the plasticity of cortical areas and white matter fibers after surgery appear worthy of investigation, so further studies could be needed to explore this topic in-depth and unify the findings obtained. Future directions should first evaluate the clinical applicability of the results obtained to stratify patients from a prognostic point of view. In addition, researchers should analyze possible differences in the reorganization of cortical areas according to the surgical approach used, carrying out the comparison of CSM patients before and after surgery and long-term follow-up after surgery for a better comprehension of functional reorganization patterns.

## Data Availability

All data generated or analyzed during this study are included in this published article.
